# *GNAQ* mutation in a patient with metastatic mucosal melanoma

**DOI:** 10.1186/1471-2407-14-516

**Published:** 2014-07-16

**Authors:** Chung-Young Kim, Dae Won Kim, Kevin Kim, Jonathan Curry, Carlos Torres-Cabala, Sapna Patel

**Affiliations:** 1 Department of Medicine, Seoul National University College of Medicine, Seoul, Korea; 2 Department of Melanoma Medical Oncology, University of Texas MD Anderson Cancer Center, Houston, TX, USA; 3 Department of Dermatopathology, University of Texas MD Anderson Cancer Center, Houston, TX, USA

**Keywords:** *GNAQ*, Mucosal melanoma, Mutation

## Abstract

**Background:**

Mucosal melanomas represent about 1% of all melanoma cases and classically have a worse prognosis than cutaneous melanomas. Due to the rarity of mucosal melanomas, only limited clinical studies with metastatic mucosal melanoma are available. Mucosal melanomas most commonly contain mutations in the gene *CKIT*, and treatments have been investigated using targeted therapy for this gene. Mutations in mucosal melanoma are less common than in cutaneous or uveal melanomas and occur in descending order of frequency as: *CKIT* (20%), *NRAS* (5%) or *BRAF* (3%). Mutations in G-alpha proteins, which are associated with activation of the mitogen-activated protein kinase pathway, have not been reported in mucosal melanomas. These G-alpha protein mutations occur in the genes *GNAQ* and *GNA11* and are seen at a high frequency in uveal melanomas, those melanomas that begin in the eye.

**Case presentation:**

A 59-year old Caucasian male was diagnosed with a mucosal melanoma after evaluation for what was thought to be a hemorrhoid. Molecular analysis of the tumor revealed a *GNAQ* mutation. Ophthalmologic exam did not disclose a uveal melanoma.

**Conclusion:**

Here we report, to our knowledge, the first known case of *GNAQ* mutation in a patient with metastatic mucosal melanoma.

## Background

The incidence and the mortality of melanoma have increased over the last several decades with more than 76,000 estimated new cases and more than 9,000 estimated deaths in the USA in 2013 [1]. Although most of melanoma is cutaneous in origin, it can arise from extra-cutaneous sites such as the uveal tract and mucosal surfaces where melanocytes exist. The mucosal origin melanomas which mostly arise from the mucosal membrane of the head and neck, the anorectal mucosa and the vulvovaginal mucosa have distinct biologic and clinical features compared with cutaneous melanomas. Mucosal melanomas are rare but very aggressive. The rate of mucosal melanoma is 2.2 cases per million per year, and the five-year survival is a mere 25% compared to over 200 cases per million and over 80% five-year survival for cutaneous melanoma [2,3]. The poor prognosis is likely due to the obscured anatomic sites and the rich lymphovascular supply of the mucosa [2]. Although the discovery of *BRAF* gene mutation and the advancement of immunotherapy in melanoma have led to the development of highly effective targeted therapy such as vemurafenib, dabrafenib, and trametinib and durable immunotherapy such as interleukin-2 and ipilimumab, the efficacy of these treatments in metastatic mucosal melanoma is not clear due to limited number of these patients included in clinical trials. Recently, several clinical trials reported promising results with targeting of *CKIT* mutation in mucosal melanoma [4-6]. *CKIT* mutations are reported in 21% of mucosal melanoma, and only patients with mucosal melanoma harboring a special subset of *CKIT* mutations such as *L576P* and *K642E* in exon 11 and 13 may have a clinical benefit from c-KIT inhibitors [7]. The role of amplification of *CKIT* and response to c-KIT inhibitors has also been studied [6,8]. Despite these advances, further workup is necessary to define the standard of care for mucosal melanoma.

GNAQ and GNA11 are alpha subunits of heterotrimeric G proteins, which couple seven transmembrane domain receptors to intracellular signaling pathways [9]. Mutations in the genes *GNAQ* and *GNA11* are critical for development and progression of uveal melanoma and are associated with activation of the mitogen-activated protein kinase (MAPK) pathway [10,11]. This same pathway is activated by oncogenic *BRAF* mutations in cutaneous melanoma [12]. Approximately, 80% of primary uveal melanomas have *GNAQ* or *GNA11* mutations. However, *GNAQ* or *GNA11* mutations have not been reported in mucosal melanoma. Here, we present a patient with metastatic mucosal melanoma harboring a classic *GNAQ* mutation.

## Case presentation

**Figure 1:**
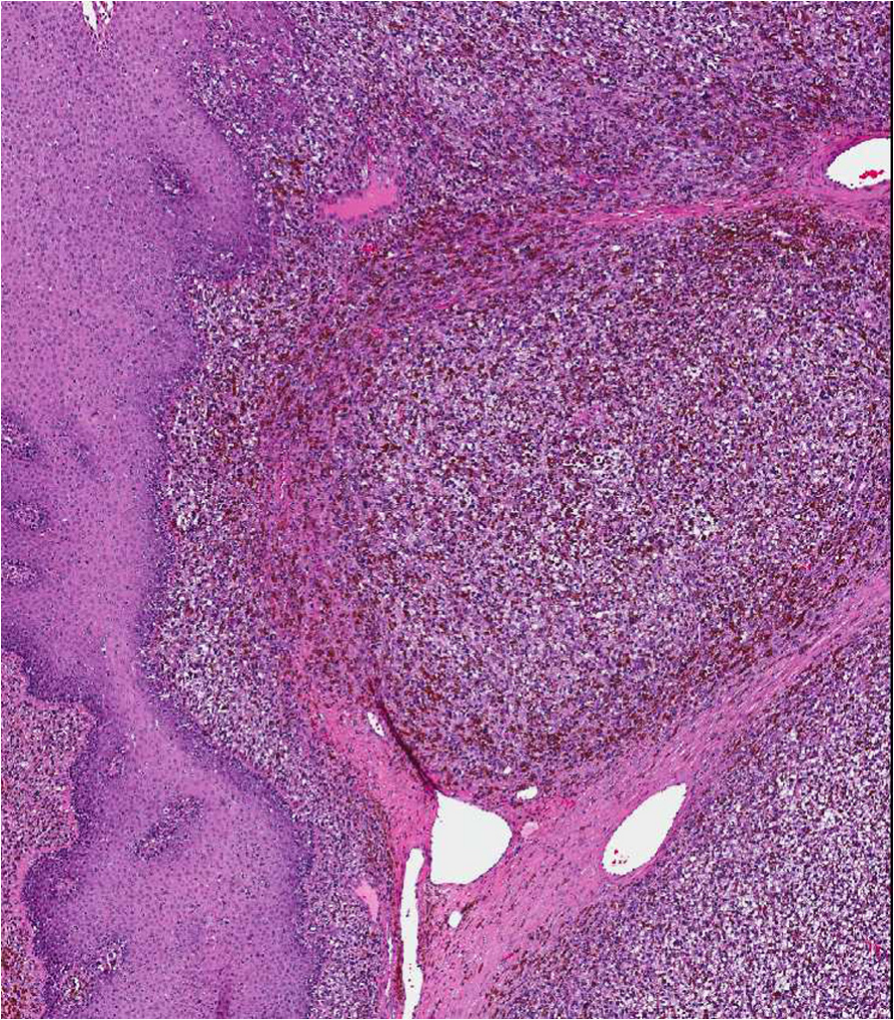
**Histolological appearance of the anal melanoma.** A bulky, polypoid mass predominantly involving lamina propria and submucosa was seen. The tumor showed focal intraepithelial lentiginous component, best interpreted as melanoma in situ (H & E, 4×).

**Figure 2:**
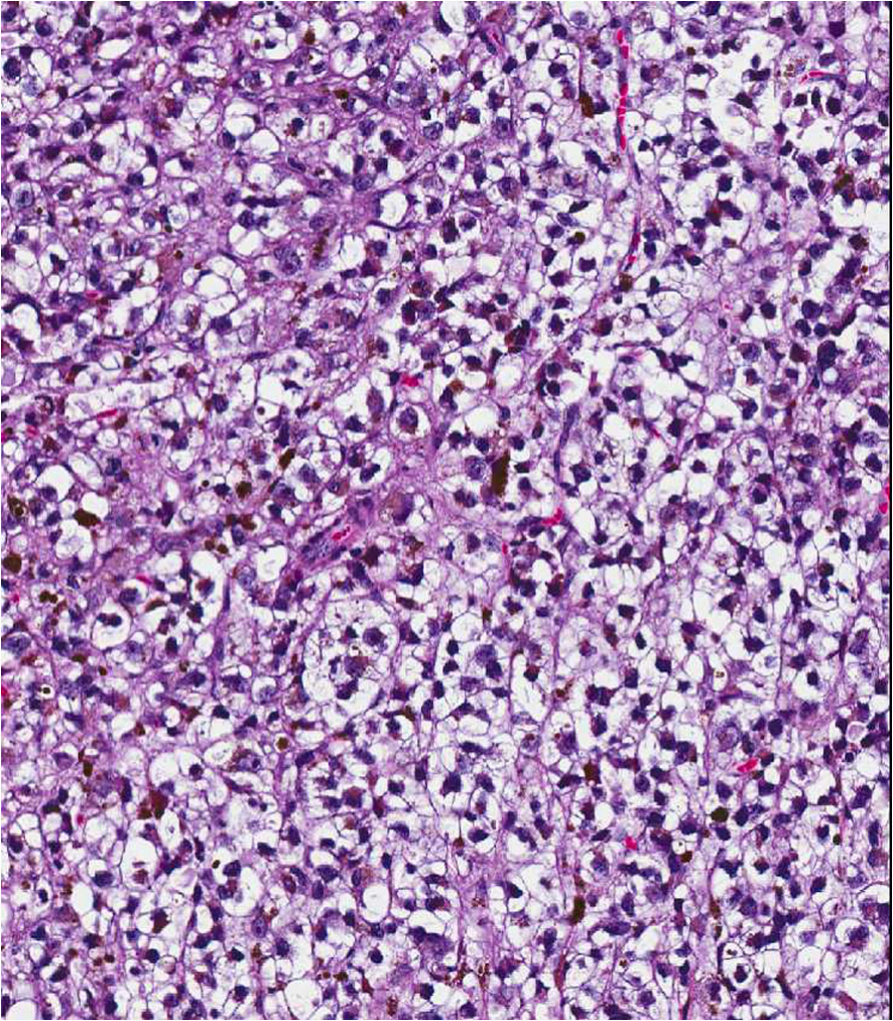
**The melanoma cells displayed diffuse clear cell change and intracytoplasmic melanin pigment (H & E, 10×).**

**Figure 3:**
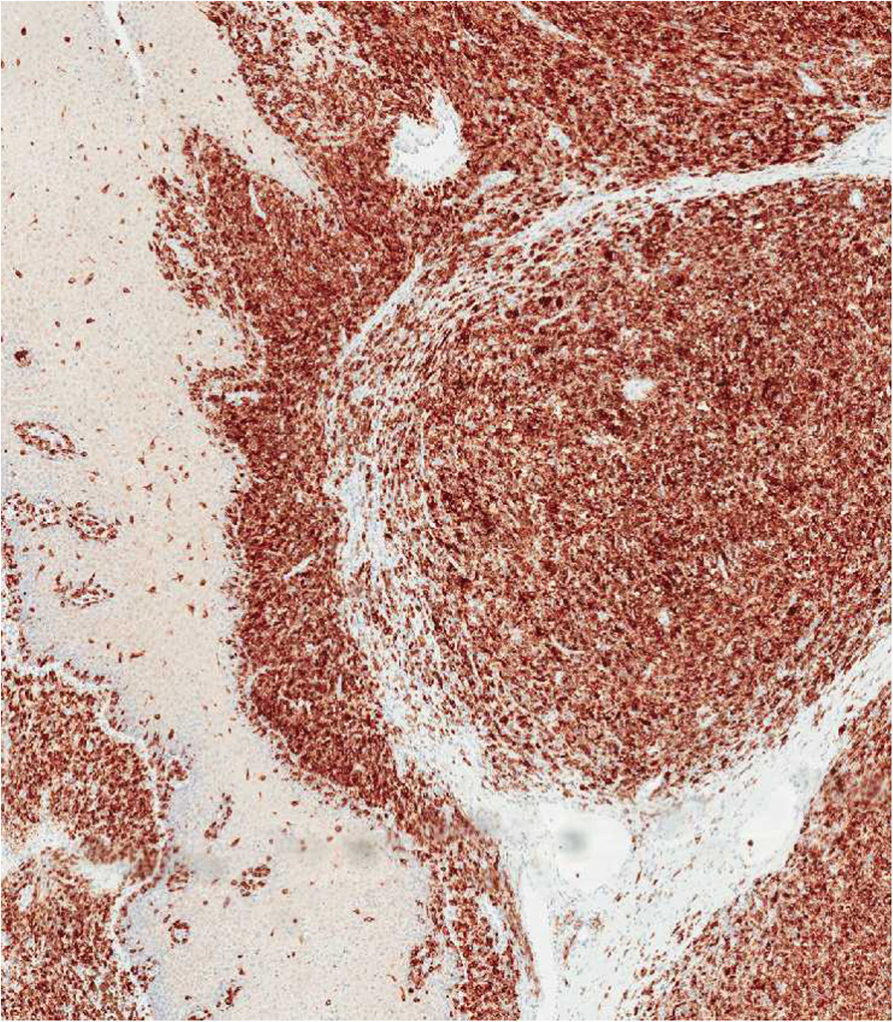
**An immunohistochemical study for MART-1 highlighted the invasive and intraepithelial components of the lesion, supporting a diagnosis of mucosal melanoma (immunohistochemical study, 4×).**

A 59-year-old otherwise healthy Caucasian man was diagnosed with a mucosal melanoma during hemorrhoid evaluation in August of 2009. Histopathological examination revealed a polypoid tumor occupying lamina propria and submucosa of the anal canal with intraepithelial lentiginous component in the center of the lesion. The tumor cells were epithelioid and showed clear cell change. Immunohistochemical studies showed the tumor cells to be positive for S100 and Melan-A. A diagnosis of a 15-mm thick mucosal melanoma with ulceration, 6 mitotic figures per mm^2^ and perineural invasion in the anal canal was made (Figures 1, 2 and 3). Molecular analysis showed the melanoma harbored a *GNAQ* mutation with wild-type *BRAF*, *KIT* and *NRAS* genes. The *GNAQ* gene mutation of the patient was the substitution of glutamine to proline in codon 209 (Q209P) which has been reported in uveal melanoma at a frequency of 20.8% but not in cutaneous melanoma or other subtypes of the disease [10,13].

The patient underwent a wide local excision of the primary melanoma with subsequent adjuvant radiation therapy. He was without relapse until January of 2010, when he had locally recurrent disease for which he underwent another wide local excision. He again remained free of disease until July of 2010 when he was found to have metastatic lesions in the perinephric lymph nodes, the liver, and lung, for which he received 2 doses of ipilimumab (3 mg/kg intravenous Day 1) and temozolomide (200 mg/m^2^ by mouth Days 1–4) [14] with further disease progression and new metastatic lesions in hilar and mediastinal lymph nodes and in the right adrenal gland. Subsequently, he received two cycles of the combination of carboplatin, paclitaxel and bevacizumab before he had further disease progression in January of 2011. He started imatinib at 400 mg twice a day in February of 2011. Due to further disease progression with imatinib, ipilimumab (3 mg/kg) was re-introduced in April of 2011 and he completed 4 cycles of ipilimumab. However, his disease progressed further with multiple metastatic lesions and he expired in August of 2011.

## Conclusions

*GNAQ* and *GNA11* mutations, which are potential drivers of MAPK activation, have been reported in blue nevi and in up to 85% of cases of uveal melanoma [10,11,15]. Mutations occur in a mutually exclusive fashion and affect codons 209 (95%) and 183 (5%) in both genes. Although *GNAQ* and *GNA11* mutations have been reported in a cutaneous melanoma case and a cell line from cutaneous melanoma [10,16], there are no data to demonstrate *GNAQ* or *GNA11* mutations in mucosal melanoma. It is possible that our patient may have had an undetected or spontaneously regressed primary uveal melanoma since his melanoma harbored the *GNAQ* mutation and metastasized to liver which is the most common metastatic site of uveal melanoma [17]. However, spontaneous regression of primary uveal melanoma is extremely rare in comparison to cutaneous melanoma [18]; furthermore, anal canal is not a common metastatic site as a single and the first metastatic lesion of uveal melanoma [17], and our patient did not have any evidence of uveal melanoma in multiple magnetic resonance image (MRI) of the brain. In addition, he had had frequent ophthalmology exams with his retina specialist due to multiple retinal detachments 3 years before he was diagnosed with melanoma. These follow-ups included slit-lamp examinations. Another possible explanation is that our patient might have an undetected or regressed primary cutaneous melanoma, since *GNAQ* mutation has been reported in one case of cutaneous melanoma [9]. However, it is less likely since our patient did not have any suspected cutaneous lesions during multiple thorough and detailed clinical examinations, and the predominant metastatic site of melanoma in the gastrointestinal tract is not the anal canal but the small bowel [19]. The melanoma seen in the anal canal, although predominantly involving lamina propria and submucosa, was interpreted to be most likely primary due to presence of intraepidermal (in situ) melanoma showing lentiginous pattern of growth (as mucosal melanoma frequently does), along with the lack of clinical evidence of tumor elsewhere at the time of diagnosis. Since *GNAQ* mutations are potential drivers of MAPK activation similar to oncogenic *BRAF*, and a recent clinical study demonstrated a significant clinical benefit of selumetinib (a selective MEK inhibitor) in metastatic uveal melanoma with *GNAQ* or *GNA11* mutations [20], our patient might have achieved clinical response with selumetinib. Unfortunately, he did not receive a MEK inhibitor as part of his treatment course.

To our knowledge, this is the first case report to demonstrate mucosal melanoma harboring a *GNAQ* mutation. This case suggests that molecular profiling may give us better understanding of genetic changes in mucosal melanoma and may afford actionable targets for therapy.

### Consent

Written informed consent was obtained from the patient’s next of kin for publication of this Case report and any accompanying images. A copy of the written consent is available for review by the Editor of this journal.

## Competing interests

The authors declare that they have no competing interests.

## Authors’ contributions

CK, DK, KK, and SP drafted the manuscript. JC and CT performed the histological review and immunoassays. SP conceived of the manuscript and carried out interpretation of molecular findings. All authors read and approved the final manuscript.

## Pre-publication history

The pre-publication history for this paper can be accessed here:

http://www.biomedcentral.com/1471-2407/14/516/prepub
